# Mutations in SACPD-C Result in a Range of Elevated Stearic Acid Concentration in Soybean Seed

**DOI:** 10.1371/journal.pone.0097891

**Published:** 2014-05-20

**Authors:** Militza Carrero-Colón, Nathan Abshire, Daniel Sweeney, Erik Gaskin, Karen Hudson

**Affiliations:** 1 Crop Production and Pest Control Research Unit, Agricultural Research Service (ARS), United States Department of Agriculture (USDA), West Lafayette, Indiana, United States of America; 2 Department of Agronomy, Purdue University, West Lafayette, Indiana, United States of America; University of Delhi South Campus, India

## Abstract

Soybean oil has a wide variety of uses, and stearic acid, which is a relatively minor component of soybean oil is increasingly desired for both industrial and food applications. New soybean mutants containing high levels of the saturated fatty acid stearate in seeds were recently identified from a chemically mutagenized population. Six mutants ranged in stearate content from 6–14% stearic acid, which is 1.5 to 3 times the levels contained in wild-type seed of the Williams 82 cultivar. Candidate gene sequencing revealed that all of these lines carried amino acid substitutions in the gene encoding the delta-9-stearoyl-acyl-carrier protein desaturase enzyme (SACPD-C) required for the conversion of stearic acid to oleic acid. Five of these missense mutations were in highly conserved residues clustered around the predicted di-iron center of the SACPD-C enzyme. Co-segregation analysis demonstrated a positive association of the elevated stearate trait with the SACPD-C mutation for three populations. These missense mutations may provide additional alleles that may be used in the development of new soybean cultivars with increased levels of stearic acid.

## Introduction

Stearic acid is one of the component fatty acids in soybean oil, comprising 2–4% of the total oil fraction. Stearic acid has a neutral effect on blood serum LDL cholesterol concentration and is therefore a desirable constituent of oils for food use [1]. Stearic acid confers a high melting temperature and oxidative stability to oils destined for end use in baking fats. Previously, to increase the proportion of stearic acid in soybean oil, the oil was subjected to hydrogenation. However, genetic manipulation of stearic acid level is more efficient and reduces the trans-fats that may be introduced by the hydrogenation process [2].

Three soybean genes have been characterized with homology to delta-9-stearoyl-acyl carrier protein desaturases (SACPDs) which are required for the conversion of stearic acid to oleic acid [3]. These genes are delimited SACPD-A, SACPD-B, and SACPD-C. SACPD-C encodes the seed-specific isoform of this enzyme, where SACPD-A and SACPD-B transcripts accumulate in all soybean tissues [1], [4].

Soybeans with mutations in the SACPD-C and SACPD-B genes have been described. FAM94-41 is a spontaneously occurring change in the SACPD-C gene and results in plants with levels of stearic acid in the seed of ∼9% [5]. Deletion of the SACPD-C gene in the A6 germplasm line results in up to 28% stearic acid in the seed, but the size of this deletion is uncharacterized [4], [6]. Additional SACPD-C mutants have been described with a range of 10-16% stearic acid in the seeds [4], [7]. SACPD-B mutants have recently been reported to contain ∼10% stearic acid [8]. No mutations have been described for the SACPD-A gene. Some high stearate mutants have previously been associated with poor germination and low seed yield [9], [10] however recently it was demonstrated that missense mutations in *SACPD-C* are not associated with poor agronomic characteristics [11]. Additional sources of germplasm carrying novel mutations in the SACPD-C gene, or novel loci which influence seed stearic acid levels are needed to circumvent this issue to enable the production of soybeans with elevated levels of stearic acid to meet the demands of the food-oil market.

## Materials and Methods

### Plants and growth conditions and fatty acid analysis

For screening, plants were grown in the field in West Lafayette, Indiana, as described in reference [12]. Field location GPS coordinates are latitude 40.468 degrees north, longitude minus 86.991 degrees west. Soybeans described in this study are non-transgenic, therefore no specific permits were required for growth. Fatty acid composition analysis was performed as previously described [13].

### Sequencing and Genotyping

Three segments of the SACPD-C (Glyma14g27990) coding region were amplified and sequenced using the primers in Table S1. DNA sample preparation for sequencing was performed using the CTAB method [14] and sample preparation for genotyping was as previously described [13]. dCAPS genotyping [15] was performed using standard protocols with the assays developed specifically for the SACPD-C mutants provided in Table S1. To evaluate the position of substitutions, mutations were overlaid on the protein structure PDB ID 1AFR using the program Cn3D v. 4.3 [16]. Mutant SACPD-C sequences are deposited in GenBank with accession numbers KJ522450-KJ522455.

## Results and Discussion

Mutant plants with high levels of stearic acid in seeds were identified in an ongoing screen for soybean seed with altered fatty acid composition (reference [12], and unpublished data) and six lines were chosen for further characterization. These mutants were obtained from an NMU-mutagenized population in the Williams-82 genetic background [17]. Levels of stearic acid in the seed of the mutant lines ranged from 6–13% (Table 1), with the highest levels in line 18190. Line #18948 corresponds to line #14, line 18190 corresponds to #16, and line 18610 corresponds to #17 in reference [12] while the isolation of lines 15073, 14197, and 21084 has not been previously described. Line 14197 and 18948 were isolated as heterozygous M_3_, as revealed by segregating types of M_4_ seed, while the remaining lines showed a consistent level of stearic acid across multiple M_4_ individuals and were presumed to be homozygous isolates (Table S2 and [12]).

**Table 1:** Elevated stearic acid levels in *SACPD-C* mutants.

	Palmitic Acid (16∶0)	Stearic Acid (18∶0)	Oleic Acid (18∶1)	Linoleic Acid (18∶2)	Linolenic Acid (18∶3)	n
W82	10.0±0.2	4±0.1	20.6±0.5	57.0±0.5	8.3±0.2	6
15073 SACPD-C_G224E_	8.1±0.4	12.1±1.2	15.9±0.6	55.0±1.3	8.8±0.6	6
	1.3e^−6^*	1.8e^−8^*	4.6e^−8^*	*0.0055* ^*^	*0.13*	
14197 SACPD-C_Y211C_	8.8±0.4	12.1±3.5	20.0±2.9	51.6±0.7	7.6±0.6	3
	1.25e^−4^*	5.3e^−4^*	*0.62*	2.4e^−6^*	0.0044*	
18190 SACPD-C_A218E_	8.5±0.6	13.5±0.6	14.8±0.2	53.8±0.4	9.3±0.3	3
	4.2e^−4^*	1.8e^−9^*	2.6e^−7^*	1.8e^−5^*	5.0e^−4^*	
18948 SACPD-C_H223R_	10.1±0.5	9.5±1.3	17.4±0.6	54.9±1.1	8.1±0.4	4
	*0.75*	4.4e^−6^*	1.3e^−5^*	0.0034*	*0.26*	
18610 SACPD-C_A239T_	9.45±0.6	6.1±1.2	22.2±1.2	54.1±2.0	8.1±0.9	6
	0.032*	0.0021*	0.0093*	0.0057*	*0.57*	
21084 SACPD-C_R329I_	8.4±0.3	8.7±0.8	27.8±1.8	48.6±1.4	6.5±0.8	4
	*0.79*	5.3e^−4^*	*0.016*	*0.51*	*0.77*	

Fatty acid levels were averaged for n homozygous M_4_ or M_5_ lines for each mutation, and averages and standard deviations are shown. *p*-value (in italics) was calculated from a two-tailed, type 2 t-test for the average fatty acid level in each mutant relative to the Williams-82 (W82) wild type control. Single asterix indicates *p*-values that are significant at the *p*<0.05 level.

**Figure 1:**
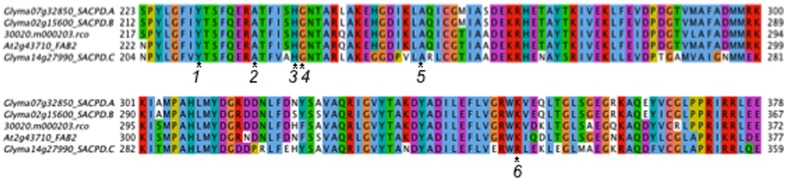
Mutations in the SACPD-C enzyme. Multiple sequence alignment for soybean SACPD-C with close sequence homologs showing the position of mutations in conserved locations. Other proteins shown are SACPD-A and SACPD-B from soybean, *FAB2* from Arabidopsis (At3g02610/DES2), and the castor bean SACPD (30020.m000203.rco). Mutations described in this work are located at the following positions: 1. SACPD-C_Y211C_ 2. SACPD-C_A218E_ 3. SACPD-C_H223R_ 4. SACPD-C_G224E_ 5. SACPD-C_A239T_ 6. SACPD-C_R329I_.

Sequencing of the SACPD-C gene from these mutant lines revealed that each carries a distinct and independent missense mutation in the coding region of SACPD-C (Figure 1 and Table 1). The sequence of the SACPD-C gene in the Williams-82 accession that serves as the germplasm background for this mutant is consistent with the previously described soybean SACPD-C sequence (Genbank #EF113911) [4] but not the Williams-82 sequence within the Phytozome database (version 9.1, www.phytozome.net) which carries a frameshift mutation early in the first exon. The exons of the SACPD-A and SACPD-B genes (Glyma07g32850 and Glyma02g15600, respectively) were also sequenced in these lines and were found to be identical to the Williams-82 sequence (not shown). As line 21084 and 18610 M_3_ and M_4_ individuals produced seed with a marginal increase in stearic acid content from ranging from 5–8%, to verify that these lines were homozygous the SACPD-C gene was sequenced from M_4_ plants (Table S2). All of these M_4_ individuals were homozygous for their respective mutations. This suggests that the mutations in these lines are less damaging to enzyme structure or function and the resultant plants display a marginal increase in stearic acid.

Visualization of the location of the affected amino acids in the determined crystal structure for the SACPD enzyme from *Ricinus communis* reveals that five of the six mutations are located in the enzyme channel domain which is composed of several alpha helixes, the function of this channel is to position the hydrocarbon chain of the fatty acid so that it can be oxidized (Figure S1) [18]. SACPD-C_Y211C_, SACPD-C_A218E_, SACPD-C_G224E_, SACPD-C_H223R_ are located on alpha helix six, and SACPD-C_A239T_ is located on alpha helix seven. The amino acid substitutions introduce either changes in side chain charge or size into this region of the enzyme. These mutations may reduce the ability of a fatty acid chain to be correctly positioned within the channel domain of the enzyme. Proximity of mutations to the di-iron core and increases in polarity also correlate with greater reduction of enzymatic function [18]. SACPD-C_R329I_ is located on a loop residue after helix ten, and this mutation results in a less dramatic increase in stearic acid level. We speculate that this residue may be involved in correct folding of the active site or possibly in interaction with other proteins.

**Figure 2:**
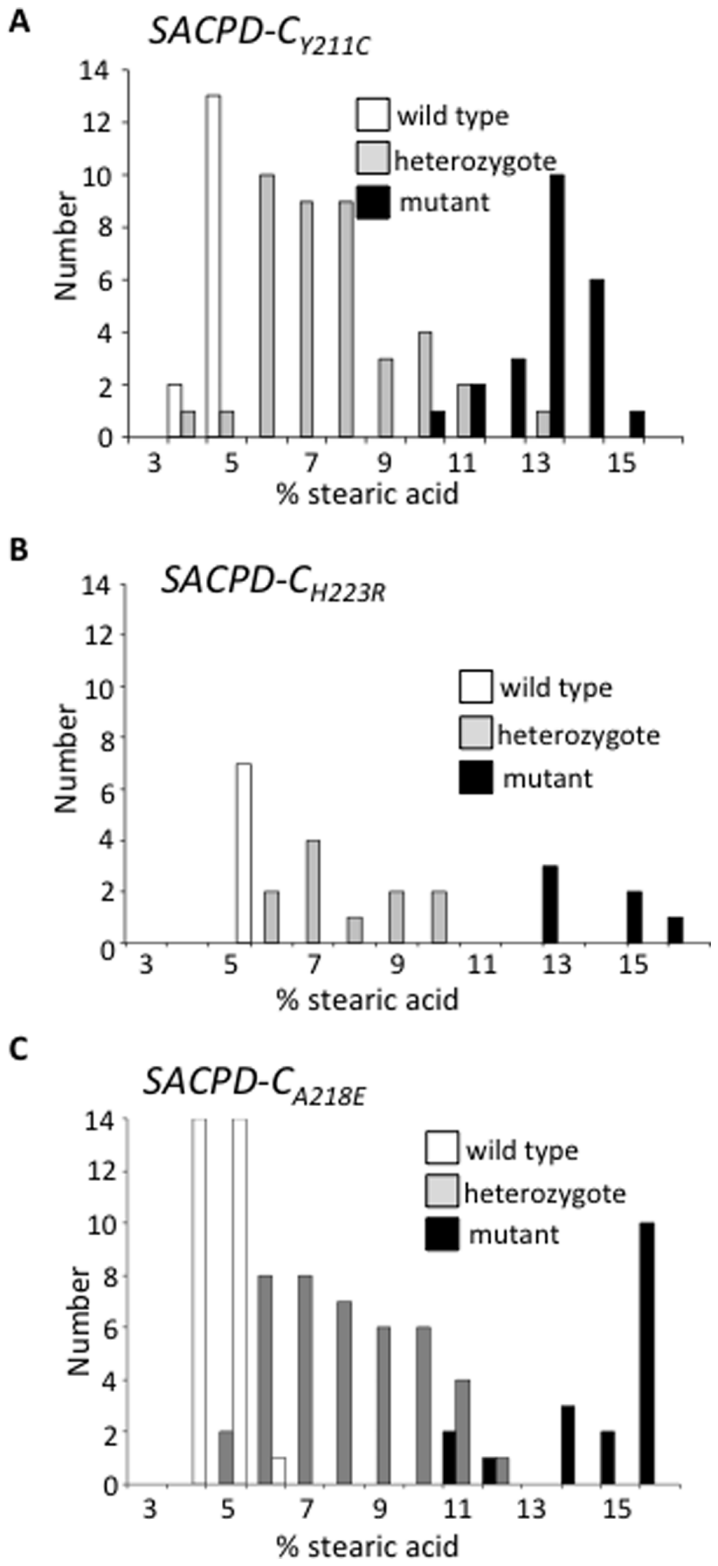
Cosegregation of SACPD-C mutations and elevated stearic acid phenotype. Individuals from segregating populations were analyzed for stearic acid content and genotyped for A. SACPD-C_Y211C_ (78 indivduals) B. SACPD-C_H223R_ (24 individuals), and C. SACPD-C_A218E_ (89 individuals). White bars indicate wild-type individuals, grey bars indicate heterozygous individuals, and black bars indicate homozygous mutants.

To demonstrate that changes in the SACPD-C gene cause the elevated stearic acid phenotype in these mutant lines, co-segregation analysis was performed in segregating populations. In each case the single nucleotide polymorphism introduced by the mutation was used to develop a codominant dCAPs marker. Lines 18948 (SACPD-C_H223R_) and 14197 (SACPD-C_Y211C_) were isolated as heterozygous mutants for SACPD-C mutations. M_4_ plants segregating for these SNPs were grown in the field during the 2011 growing season, and M_5_ seed was genotyped for the SNP and phenotyped for fatty acid content. Figure 2A shows association of the SACPDC_Y211C_ mutation with elevated levels of stearic acid in the seed. Figure 2B shows cosegregation of SACPDC_H223R_ with elevated levels of stearic acid in the seed. Line 18190 (SACPD-C_A218E_), which has the highest levels of stearate, was crossed to the cultivar Prize, allowed to self-pollinate, and F_2_ plants were grown in the field (during the 2012 growing season) and genotyped. F_3_ seed from F_2_ individuals was analyzed for stearic acid content. Figure 2C shows the cosegregation of SACPDC_A218E_ with elevated levels of stearate. In the SACPDC_A218E_ x Prize segregating population, a number of homozygous mutant individuals were observed which contained stearic acid levels greater (>20% stearate) than those observed in the homozygous mutant isolate in the Williams-82 background (approximately 13%). This may be due to the segregation of a second, modifying locus distinct from that of the mutated SACPD-C gene in the SACPD-C_A218E_ line. This genetic modifier may be in the outcross parent (Prize) genetic background, or it may be a second-site mutation in the heavily mutagenized background of the SACPD-C_A218E_ parent line. It is estimated that the mutation frequency in the parent of SACPD-C_A218E_ parent is on the order of 100 genic mutations per individual [17]. The seed stearate level in the Prize parent grown in parallel was 4%, similar to Williams-82. As the other SACPD genes would be good candidates to act additively with SACPD-C_A218E_, the SACPD-A, -B, (and –C) genes were sequenced in the Prize parent and found to encode predicted proteins with an amino acid sequence identical to the published reference sequence [4].

There is evidence that other, unknown loci in addition to SACPD-C impact stearic acid levels in soybean seed [8]. The strong mutations in SACPD-C described here are comparable to the defects observed in RG7 and RG8 which are also chemically induced point mutants in SACPD-C [7] and may therefore serve as a basis for germplasm to enhance stearic acid levels. Weaker alleles such as SACPD-C_R329I_ and SACPD-C_A239T_ may be useful when combined with other genes to increase stearic acid while maintaining high oleic acid levels. In addition, identification of the factors that enable the >20% stearic acid levels observed in association with SACPD-C_A218E_ may suggest to further increase stearic acid levels for a soybean oil with enhanced functionality.

## Supporting Information

Figure S1 **Position of mutations in SACPD-C crystal structure.**
(TIFF)

Table S1 **Primer sequences.**
(PDF)

Table S2 **M_3/_M_4_ phenotypes and genotypes.**
(PDF)

## References

[1] Byfield GE, Xue H, Upchurch RG (2006) Two Genes from Soybean Encoding Soluble Δ9 Stearoyl-ACP Desaturases. Crop Sci 46: 840–846.

[2] Clemente TE, Cahoon EB (2009) Soybean Oil: Genetic Approaches for Modification of Functionality and Total Content. Plant Physiology 151: 1030–1040. 19783644 10.1104/pp.109.146282 PMC2773065

[3] Ohlrogge J, Browse J (1995) Lipid biosynthesis. Plant Cell 7: 957–970. 7640528 10.1105/tpc.7.7.957 PMC160893

[4] Zhang P, Burton JW, Upchurch RG, Whittle E, Shanklin J, et al (2008) Mutations in a Delta(9)-Stearoyl-ACP-Desaturase Gene Are Associated with Enhanced Stearic Acid Levels in Soybean Seeds. Crop Science 48: 2305–2313.

[5] Pantalone VR, Wilson RF, Novitzky WP, Burton JW (2002) Genetic regulation of elevated stearic acid concentration in soybean oil. Journal of the American Oil Chemists Society 79: 549–553.

[6] Hammond EG, Fehr WR (1983) Registration of A6-Germplasm Line of Soybean. Crop Science 23: 192–193.

[7] Boersma JG, Gillman JD, Bilyeu KD, Ablett GR, Grainger C, et al (2012) New Mutations in a Delta-9-Stearoyl-Acyl Carrier Protein Desaturase Gene Associated with Enhanced Stearic Acid Levels in Soybean Seed. Crop Sci 52: 1736–1742.

[8] Ruddle P 2nd, Whetten R, Cardinal A, Upchurch RG, Miranda L (2013) Effect of a novel mutation in a Delta9-stearoyl-ACP-desaturase on soybean seed oil composition. Theor Appl Genet 126: 241–249. 22961205 10.1007/s00122-012-1977-5

[9] Lundeen PO, Fehr WR, Hammond EG, Cianzio SR (1987) Association of Alleles for High Stearic-Acid with Agronomic Characters of Soybean. Crop Science 27: 1102–1105.

[10] Rahman SM, Takagi Y, Kinoshita T (1997) Genetic control of high stearic acid content in seed oil of two soybean mutants. Theoretical and Applied Genetics 95: 772–776.

[11] Ruddle P, Cardinal A, Upchurch RG, Arellano C, Miranda L (2013) Agronomic Effects of Mutations in Two Soybean Δ9–Stearoyl-Acyl Carrier Protein-Desaturases. Crop Sci 53: 1887–1893.

[12] Hudson K (2012) Soybean Oil-Quality Variants Identified by Large-Scale Mutagenesis. International Journal of Agronomy 2012: Article ID 569817, 7.

[13] Head K, Galos T, Fang Y, Hudson K (2012) Mutations in the soybean 3-ketoacyl-ACP synthase gene are correlated with high levels of seed palmitic acid. Molecular Breeding 30: 1519–1523.

[14] Richards E, Reichardt M, Rogers S (2001) Preparation of Genomic DNA from Plant Tissue. Current Protocols in Molecular Biology: John Wiley & Sons, Inc. 10.1002/0471142727.mb0203s27 18265183

[15] Neff MM, Neff JD, Chory J, Pepper AE (1998) dCAPS, a simple technique for the genetic analysis of single nucleotide polymorphisms: experimental applications in Arabidopsis thaliana genetics. Plant J 14: 387–392. 9628033 10.1046/j.1365-313x.1998.00124.x

[16] CN3D http://www.ncbi.nlm.nih.gov/Structure/CN3D/cn3d.shtml

[17] Cooper JL, Till BJ, Laport RG, Darlow MC, Kleffner JM, et al (2008) TILLING to detect induced mutations in soybean. BMC Plant Biol 8: 9. 18218134 10.1186/1471-2229-8-9 PMC2266751

[18] Lindqvist Y, Huang W, Schneider G, Shanklin J (1996) Crystal structure of delta9 stearoyl-acyl carrier protein desaturase from castor seed and its relationship to other di-iron proteins. EMBO J 15: 4081–4092. 8861937 PMC452130

