# Supplementary figures and images for: Mutations in SACPD-C Result in a Range of Elevated Stearic Acid Concentration in Soybean Seed
Source: PLoS One. 2014 May 20;9(5):e97891. doi: 10.1371/journal.pone.0097891 (PMC4028252; doi:10.1371/journal.pone.0097891)

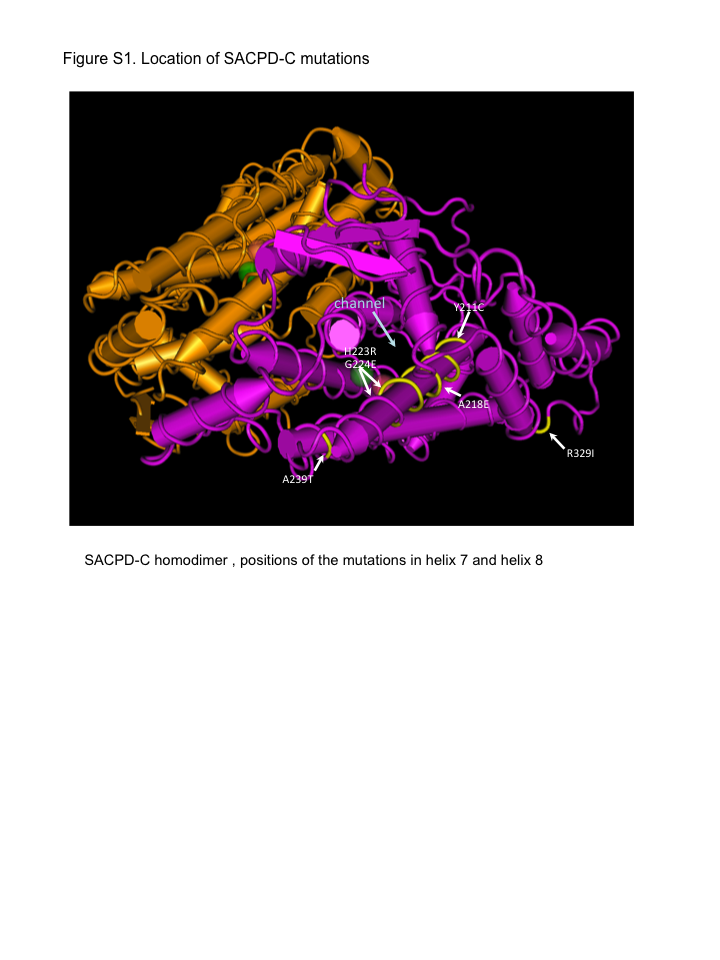

Supplement: Figure S1 — Position of mutations in SACPD-C crystal structure. (TIFF) [file pone.0097891.s001.tiff]
