# Supplementary material for: Mutations in SACPD-C Result in a Range of Elevated Stearic Acid Concentration in Soybean Seed
Source: PLoS One. 2014 May 20;9(5):e97891. doi: 10.1371/journal.pone.0097891 (PMC4028252; doi:10.1371/journal.pone.0097891)
Supplement: Table S1 — Primer sequences. (PDF) [file pone.0097891.s002.pdf]

Supplemental Table 1. Sequencing and genotyping primers

Genotyping primers

Name	Sequence	Purpose	Mutation	enzyme (for dCAPS marker)
kk505	TGTATTTTGTTTTGATTAAATTGGGTA	genotyping	14197/SACPDC_Y211C	
kk532	CCACGATTCTTGAGTACGCGTTC	genotyping (dCAPS)	14197/SACPDC_Y211C	RsaI
kk508	CCTGGTTTCACGTGACATGG	genotyping	18948/SACPDC_H223R	
kk559	CCTTCGCGAGCCGAGCCGTCACCCC	genotyping (dCAPS)	18948/SACPDC_H223R	DraIII
kk689	GCCGAGCCGTGTTCCCGTGCGCCGCAAATGTT	genotyping (dCAPS)	18190/SACPDC_A218E	BstAP I
kk690	TGGCAATCGGAGCTTTCTCATAG	genotyping	18190/SACPDC_A218E	

Sequencing primers

Name	Sequence	Location	Purpose
kk655	ACGTACCAAAACCACAGCAC	SACPD-C UTR+exon1	amplify +sequence
kk656	GGGAGGAAGTTTTGTGGCTG	SACPD-C UTR+exon2	amplify +sequence
kk497	TCAAAACCATGCAGATACGAAC	SACPD-C exon 2	amplify +sequence
kk504	TTTTGCTTCATGTGGAATGG	SACPD-C exon 2	amplify +sequence
kk690	TGGCAATCGGAGCTTTCTCATAG	SACPD-C exon3	amplify +sequence
kk509	CAACAACCATGGATCCAACA	SACPD-C exon4	amplify +sequence
kk649	CCTTCTTCTTCTGCCTTACATC	SACPD-A exon 1	amplify+sequence
kk650	TTAGACACTCAACCACAC	SACPD-A exon 1	amplify+sequence
kk493	TGTTGTACATGTGGGGGCTA	SACPD-A exon 2/3	amplify
kk640	GCGTATACAAATATATGAACATAGTAACAAACC	SACPD-A exon 2/3	amplify
kk494	ACTACCATTGCGGAAGACCA	SACPD-A exon 2/3	sequence only
kk639	GCTGCTGCTTTTGTCACTTTTTG	SACPD-A exon 2/3	sequence only
kk645	CAAACTGAATTTTCAACTATTC	SACPD-A exon 2/3	sequence only
kk641	AAAGGGGAGAGTGGTTTGTGAAG	SACPD-B exon 1	amplify+sequence
kk642	CTCCTTCTACATTACTCTCTCTTCTCC	SACPD-B exon 1	amplify+sequence
kk636	GATGCCATGCAAACTTCATGTTC	SACPD-B exon 2/3	amplify
kk637	CCCAGCAAGATTACATGTAATCAGTTGAC	SACPD-B exon 2/3	amplify
kk638	TGCTGCACTTATCACATTTCTG	SACPD-B exon 2/3	sequence only
kk645	CAAACTGAATTTTCAACTATTC	SACPD-B exon 2/3	sequence only
kk646	AAAACTACAATAGCAGAAGACC	SACPD-B exon 2/3	sequence only
