# Supplementary material for: Mutations in SACPD-C Result in a Range of Elevated Stearic Acid Concentration in Soybean Seed
Source: PLoS One. 2014 May 20;9(5):e97891. doi: 10.1371/journal.pone.0097891 (PMC4028252; doi:10.1371/journal.pone.0097891)
Supplement: Table S2 — M3/M4 phenotypes and genotypes. (PDF) [file pone.0097891.s003.pdf]

Supplemental Table 2. M3 and M4 phenotypes and genotypes

	% palmitic acid	% stearic acid	% oleic acid	%linoleic acid	% linolenic acid	Genotype
14197 M3	8.8	15.0	18.2	49.7	8.2	ND
14197 M4 lines, field 2010						
14197.1	10.5	9.5	20.3	52.0	7.6	ND
14197.3	10.1	8.5	20.3	53.2	8.0	MUTANT
14197.4	11.0	4.6	24.2	53.2	7.0	WT
14197.5	10.7	4.3	24.8	53.1	7.1	WT
14197.6	11.2	3.9	25.3	52.8	6.8	WT
14197.7	9.8	9.2	25.4	48.5	7.1	HETEROZYGOTE
14197.8	11.4	4.2	24.7	52.5	7.2	WT
14197.9	10.4	5.8	25.3	51.8	6.8	ND
14197.10	11.3	4.1	22.9	54.2	7.5	WT
14197.11	9.1	14.6	17.4	50.9	8.1	ND
14197.12	11.0	4.4	29.9	48.7	6.0	WT
14197.13	10.4	4.3	23.8	54.1	7.5	ND
14197.14	12.1	4.0	19.9	55.7	8.3	ND
14197.15	10.1	9.3	20.0	52.1	8.7	ND
14197.16	10.5	5.3	25.5	51.9	6.8	HETEROZYGOTE
14197.17	9.8	4.1	27.0	51.9	7.3	WT
14197.18	10.0	7.9	21.9	52.6	7.6	HETEROZYGOTE
14197.19	9.9	8.9	21.1	52.1	8.0	HETEROZYGOTE
14197.20	10.7	3.8	25.1	53.4	7.0	WT
14197.22	9.8	4.6	28.9	50.1	6.6	WT
						(by genotyping assay)
18948 M3	10.9	10.1	19.2	51.7	8.1	ND
18948 M4 lines, field 2009						(presumed segregating)
18948.1	11.2	3.4	20.3	56.6	8.6	
18948.2	10.1	8.4	16.8	56.1	8.6	
18948.3	10.6	9.0	17.3	55.2	7.9	
18948.4	10.4	3.5	21.9	56.4	7.7	
18948.5	10.4	3.3	21.6	56.5	8.3	
18948.6	10.5	7.5	17.5	56.6	8.1	

18948.7	11.2	6.7	19.2	55.9	7.1	
18948.8	10.0	3.9	22.0	56.2	8.0	
18948.9	10.1	8.8	17.2	55.6	8.4	
18948.10	10.4	3.4	20.8	57.7	7.8	
18948.11	10.3	9.3	17.1	55.1	8.2	
18948.12	10.5	3.9	20.9	57.1	7.7	
18948.13	10.3	3.7	21.9	56.7	7.5	
18948.14	10.9	3.7	20.6	58.1	6.8	
18948.15	9.4	11.3	18.2	53.4	7.7	
21084 M3	8.4	8.7	27.8	48.6	6.5	ND
21084 M4 lines, field 2012						
21084.1	10.0	5.9	20.7	55.6	7.9	MUTANT
21084.3	10.0	5.0	16.8	58.6	9.4	MUTANT
21084.4	10.3	5.5	16.8	58.5	8.8	MUTANT
21084.7	9.6	6.9	18.8	57.0	7.6	MUTANT
						(by sequencing)
18610 M3	10.22	7.12	25.02	50.96	6.68	ND
18610 M4, field 2012						
18610.1	9.7	5.2	20.2	56.6	8.2	MUTANT
18610.2	9.6	6.4	21.1	54.6	8.2	MUTANT
18610.3	9.6	6.1	21.6	55.6	7.1	MUTANT
18610.4	9.4	7.1	21.7	53.7	8.1	MUTANT
18610.5	9.4	5.9	20.4	56.0	8.2	MUTANT
18610.6	9.3	5.9	19.8	57.1	8.0	MUTANT
18610.7	9.3	6.3	20.1	55.7	8.5	MUTANT
18610.8	9.7	6.2	20.9	55.2	8.0	MUTANT
18610.9	9.6	5.9	21.3	55.5	7.8	MUTANT
18610.10	9.6	5.5	20.0	56.9	8.0	MUTANT
18610.11	9.5	6.3	20.3	56.2	7.8	MUTANT
18610.12	9.4	5.9	21.6	55.5	7.7	MUTANT
18610.13	9.6	6.0	21.2	55.2	8.0	MUTANT

						(by sequencing)
15073 M3	8.14	15.19	18.15	50.35	8.17	ND
15073 M4, field 2010						presumed homozygous
15073.1	8.88	10.02	16.11	56.06	8.93	
15073.2	8.70	10.84	18.67	53.51	8.28	
15073.3	8.48	11.27	16.29	55.40	8.56	
15073.4	8.51	11.72	17.00	54.13	8.63	
15073.5	8.87	10.60	17.20	54.63	8.71	
15073.6	8.38	11.46	17.86	53.49	8.81	
15073.7	8.51	12.25	16.37	54.47	8.40	
15073.8	7.82	12.84	17.45	53.18	8.71	
15073.9	8.56	13.58	17.44	52.46	7.96	
15073.10	8.08	12.58	16.87	54.55	7.92	
15073.11	8.14	12.89	17.47	52.97	8.53	
15073.12	8.25	13.53	17.28	53.22	7.72	
18190 M3	8.61	15.32	17.15	50.91	8.01	ND
18190 M4, field 2008						presumed homozygous
18190.1	8.79	16.73	16.52	49.65	8.31	
18190.2	8.97	13.71	16.76	51.89	8.68	
18190.3	8.48	18.13	17.43	47.87	8.09	
18190.4	10.38	4.19	23.55	53.68	8.22	
18190.5	9.19	14.15	17.25	50.89	8.52	
18190.6	9.21	16.98	16.80	48.52	8.49	
18190.7	8.68	15.55	16.93	50.59	8.25	
18190.8	8.57	12.64	17.42	52.25	9.11	
18190.9	8.73	15.09	17.84	50.49	7.85	
18190.10	9.21	15.57	17.38	49.42	8.42	

ND - not determined

Genotyping assays, where available, or Sanger di-deoxy sequencing was used where indicated to determine the genotype of M4 individuals.
